# Preoperative tumor marking with indocyanine green (ICG) prior to minimally invasive colorectal cancer: a systematic review of current literature

**DOI:** 10.3389/fsurg.2023.1258343

**Published:** 2023-08-11

**Authors:** Michael K. Konstantinidis, Argyrios Ioannidis, Panteleimon Vassiliu, Nikolaos Arkadopoulos, Ioannis S. Papanikolaou, Konstantinos Stavridis, Gaetano Gallo, Dimitrios Karagiannis, Manish Chand, Steven D. Wexner, Konstantinos Konstantinidis

**Affiliations:** ^1^ Department of General, Laparoscopic, Oncologic and Robotic Surgery, Athens Medical Center, Athens, Greece; ^2^ Fourth Department of Surgery, Attikon University Hospital, National and Kapodistrian University of Athens School of Medicine, Athens, Greece; ^3^ Hepatogastroenterology Unit, Second Department of Internal Medicine—Propaedeutic, Medical School, National and Kapodistrian University of Athens, Attikon University General Hospital, Athens, Greece; ^4^ 2nd Department of Obstetrics and Gynaecology, Aretaieion Hospital, University of Athens, Athens, Greece; ^5^ Department of Surgical Sciences, Sapienza University of Rome, Rome, Italy; ^6^ Department of Gastroenterology and Hepatology, Athens Medical Center, Athens, Greece; ^7^ UCL Division of Surgery and Interventional Sciences, WEISS Centre, University College London, London, United Kingdom; ^8^ Department of Colorectal Surgery, Ellen Leifer Shulman and Steven Shulman Digestive Disease Center, Cleveland Clinic Florida, Weston, FL, United States

**Keywords:** fluorescence imaging, indocyanine green, colorectal surgery, colorectal cancer, colorectal tumor, preoperative tumor marking, preoperative tattoo

## Abstract

**Aim:**

To describe the currently available evidence regarding the efficacy and safety of preoperative tumor marking using indocyanine green (ICG) prior to laparoscopic or robotic colorectal resections.

**Methods:**

A systematic search for relevant studies was conducted using the following databases: Embase (OVID), MEDLINE® (OVID), APA PsycInfo (OVID), Global Health (OVID) and HMIC Health Management Information Consortium (OVID) through June 2022 reported according to PRISMA 2020 guidelines. Primary outcome was the detection rate of the tumor sites preoperatively marked with ICG. Secondary outcomes were timing of ICG injection in days prior to the operation and technique-related complications.

**Results:**

Eight single center studies, published between 2008 and 2022, were identified yielding a total of 1,061 patients, of whom 696 were preoperatively tattooed with ICG. Injection dosage of diluted ICG ranged from 0.1–1.5 ml. Four studies used the saline test injection method prior to ICG injection. When the marking was placed within one week, the visualization rate was 650/668 (97%), whereas when it was longer than one week, the detection rate was 8/56 (14%). No severe complications were reported.

**Conclusion:**

Preoperative tumor marking using ICG prior to minimally invasive colorectal resections is safe and effective, allowing intraoperative tumor site location when performed up to a week prior to surgery without disturbing the surgical view in potential mild complications.

## Introduction

Indocyanine green (ICG) is a water-soluble fluorescent dye with binding affinity for plasma proteins, particularly lipoproteins and exhibits fluorescence within the near-infrared (NIR) spectrum (750–950 nm). Originally developed by Kodak Laboratories in 1955 for NIR photography, ICG obtained Food and Drug Administration (FDA) approval for human use in 1959 (1). Since then, it has found several applications as an imaging modality in various medical fields, including cardiac output determination as well as identification and assessment of ophthalmic angiography, hepatic function, liver and bowel blood flow, and cholangiography (2–4).

ICG administration routes include intravenous, interstitial (submucosa or subserosa) οr intra-ureteric. Following intravenous injection, the dye undergoes hepatic metabolism and is exclusively excreted into the bile, with its half-life dependent on liver function, typically ranging from 3 to 4 min (5). In cases where direct intravenous administration is not employed, ICG follows lymphatic drainage. The time required for ICG to reach the nearest lymph node is approximately 15 min (6).

The applications of ICG in colorectal surgery are extensive and continually evolving. ICG has been utilized for perfusion assessment, intraoperative ureter visualization, identification of sentinel nodes, and bowel visualization of lymphatic drainage in colorectal operations. Additionally, it has been described as a localization marker and intraoperative evaluation of peritoneal and hepatic metastases (7, 8).

Preoperative tumor marking with ICG has emerged as a promising tool in colorectal surgery. Studies evaluating preoperative tumor marking with ICG have demonstrated promising results, showcasing its potential to improve surgical outcomes. Enhanced visualization and precise tumor localization lead to increased resection accuracy, reduced positive margin rates, and decreased local recurrence rates. Additionally, ICG-guided lymph node mapping aids in determining lymph node status, allowing for more tailored treatment decisions. Ongoing research aims to refine techniques, optimize timing, dosage, and injection methods, and explore potential combination strategies with other imaging modalities. Furthermore, the integration of robotic surgery and fluorescence-guided techniques holds promise in further enhancing the accuracy and efficiency of tumor localization and resection. However, challenges such as limited depth penetration and potential adverse effects warrant caution. In this review, we critically assessed the existing body of literature regarding preoperative ICG tumor marking (tattoo) prior to colorectal operations, identified gaps in knowledge, and proposed future research directions to optimize its utilization in colorectal surgery.

## Materials and methods

### Search strategy and selection criteria

A systematic literature review was conducted according to the Preferred Reporting Items for Systematic Reviews and Meta-Analyses (PRISMA) guidelines (9). We performed an independent literature search for relevant studies from inception up to June 4, 2023, across five databases: Embase (OVID), MEDLINE® (OVID), APA PsycInfo (OVID), Global Health (OVID) and HMIC Health Management Information Consortium (OVID). The following search term was used in OVID: (colorectal surgery or colon surgery or colorectal tumor or colorectal lesions).mp AND (ICG or indocyanine green or preoperative colonoscopic marking or fluorescent guided surgery or tattooing or tattoo or endoscopic marking or tumor localization).mp. No geographical or age restrictions were applied.

### Inclusion and exclusion criteria

Randomized controlled trials (RCTs), cohort studies (prospective or retrospective) and case-control studies reporting the use of preoperative colorectal tumor marking with ICG regarding the association between injection time and detection rate were included. Furthermore, restrictions included English language and human studies.

Studies reporting patient numbers below 10 are excluded. Reviews, case reports, editorials, viewpoints, letters to the editor, and conference abstracts are excluded.

### Data extraction

After removing duplicates, publications were screened by title and abstract, then full texts were appraised to determine their eligibility by two independent authors (MK, KS). The full manuscripts of studies that met our inclusion criteria were obtained. In cases where reviewers disagreed on study eligibility, this was resolved by discussion with a third author (AI). Data from each article were extracted by one author (MK) and re-validated by another (KS). The following data were collected for each study: first author, publication year, country, study type, sample size (post-dropouts), operation type, primary endpoints (Table 1). Furthermore, procedure characteristics were collected for each study as following: ICG solution, N/S elevation, injection dosage, injection sites, injection time, and tumor location (Table 2).

**Table 1:** Characteristics of included studies.

Study	Country, year	Study type	Number of patients (ICG use)	Operation type (patients)	Primary endpoint
Satoyoshi et al.	Japan, 2020	Prospective case-series	165 (165)	Laparoscopic Colorectal Resections (165)	Intraoperative detection rate of ICG marking
Kim et al.	South Korea, 2020	Retrospective cohort	227 (90)	Laparoscopic Colorectal Resections (152) Open (75)	Compare efficacy and safety between direct injection method with ICG and saline injection method with India Ink
Sang Lee et al.	South Korea, 2018	Retrospective case-series	174 (174) tattoo sites:184	Laparoscopic Colorectal Resections (174)	1. Usefulness of preoperative colonoscopic ICG tattoo with N/S elevation 2. Visualization rates in different sites of colon that are marked within 2 days prior of surgery
Watanabe et al.	Japan, 2017	Prospective case-series	80 (80)	Laparoscopic Colorectal Resections (80)	Visibility of PINPOINT system for intraoperative identification of marked tumors with ICG
Jae Park et al.	South Korea, 2018	Retrospective cohort	342 (114)	Laparoscopic Colorectal Resections (342)	1. Usefulness of preoperative colonoscopic ICG tattoo in general prior of colorectal resections 2. Usefulness of preoperative colonoscopic ICG tattoo in groups classified according to stage and type of surgery
Nagata et al.	Japan, 2016	Prospective case-series	24 (24)	Laparoscopic Colorectal Resections (24)	Feasibility and safety of imaging method using LED-activated ICG fluorescence for laparoscopic colorectal surgery
Miyoshi et al.	Osaka, 2008	Retrospective case-series	39 (39)	Laparoscopic Colorectal Resections (N/A) Open Colorectal Resections (N/A)	Timing of ICG tattoo prior of colorectal resections
Konstantinidis et al.	Athens, 2022	Prospective case-series	10 (10)	Robotic Colorectal Resections (10)	Usefulness of preoperative colonoscopic ICG tattoo prior of robotic colorectal resections

ICG, Indocyanine green; N/S, Normal saline.

**Table 2:** Procedure characteristics of studies.

Study	ICG solution (mg/ml)	N/S elevation	Injection dosage (ml)	Injection sites	Injection time (days)	Groups of patients	Tumor location or marking sites
Satoyoshi et al.	5	Yes (0.2 ml−2 sites)	0.1	2	≤6 7–9 ≥10	Day of marking prior of operation: patients a. ≤6: 141 b. 7–9: 10 c. ≥10: 4	Right colon: 41 Left colon: 55 Rectum: 69
Kim et al.	N/A	No	0.5	3 (circumferential)	≤3	Marked with ICG or India Ink: patients a. India Ink: 79 b. ICG: 149	Right colon: 53 (ICG:42) Left colon: 127 (ICG: 68) Rectum: 57 (ICG: 39)
Sang Lee et al.	N/A	Yes (1 ml−1 site)	1–1.5	2, 180° apart or 3, 120° apart	0 1 2 3–14	Day of marking prior of operation: tattoo sites a. ≤2: 179 b. >2: 5	Ascending colon: 7 Hepatic flexure: 11 Transverse colon: 20 Splenic flexure: 5 Descending colon: 12 Sigmoid: 87 Rectosigmoid: 26 Rectum: 16
Watanabe et al.	2.5	N/A	0.5	N/A	1 3 5 6 7 8–10	Day of marking prior of operation: patients a. ≤7: 76 b. 8–17: 4	Right colon: 19 Left colon: 27 Rectum: 34
Jae Park et al.	12.5	Yes (1–2 ml)	0.5–1	4	1	Marked with ICG or India Ink: patients Yes: 114 No: 228	Right colon: 25 Left colon: 10 Rectum: 53
Nagata et al.	2.5	N/A	0.5	1 site with ICG 1 site with India Ink	≤3	Single group of patients with simultaneous tattoo with ICG and India Ink	Ascending colon: 6 Transverse colon: 3 Descending colon: 3 Sigmoid: 4 Rectosigmoid: 5 Rectum: 3
Miyoshi et al.	12.5	Yes (2 ml−1 site)	1 ml	2	1–73 (median:4)	Day of marking prior of operation: patients a. ≤8: 29 b. >9: 10	Cecum: 1 Ascending colon: 8 Transverse colon: 6 Descending colon: 2 Sigmoid: 15 Rectosigmoid: 5 Upper Rectum: 3 Lower Rectum: 1
Konstantinidis et al.	2.5	No	0.1	2	1	Single group of patients marked with ICG	Right colon: 1 Left colon: 6 Rectum: 3

ICG, Indocyanine green; N/S, Normal saline.

### Outcomes

The reported primary endpoint is the detection rate of the tumor sites preoperatively marked with ICG. The reported secondary outcomes were timing of ICG injection in days prior to the operation and technique-related complications.

### Quality assessment

The quality of the included studies was assessed by two independent reviewers (MK, KS) using the ROBINS-I tool for non-randomized studies (Figure 2) (10).

## Results

### Characteristics of studies and patients

**Figure 1:**
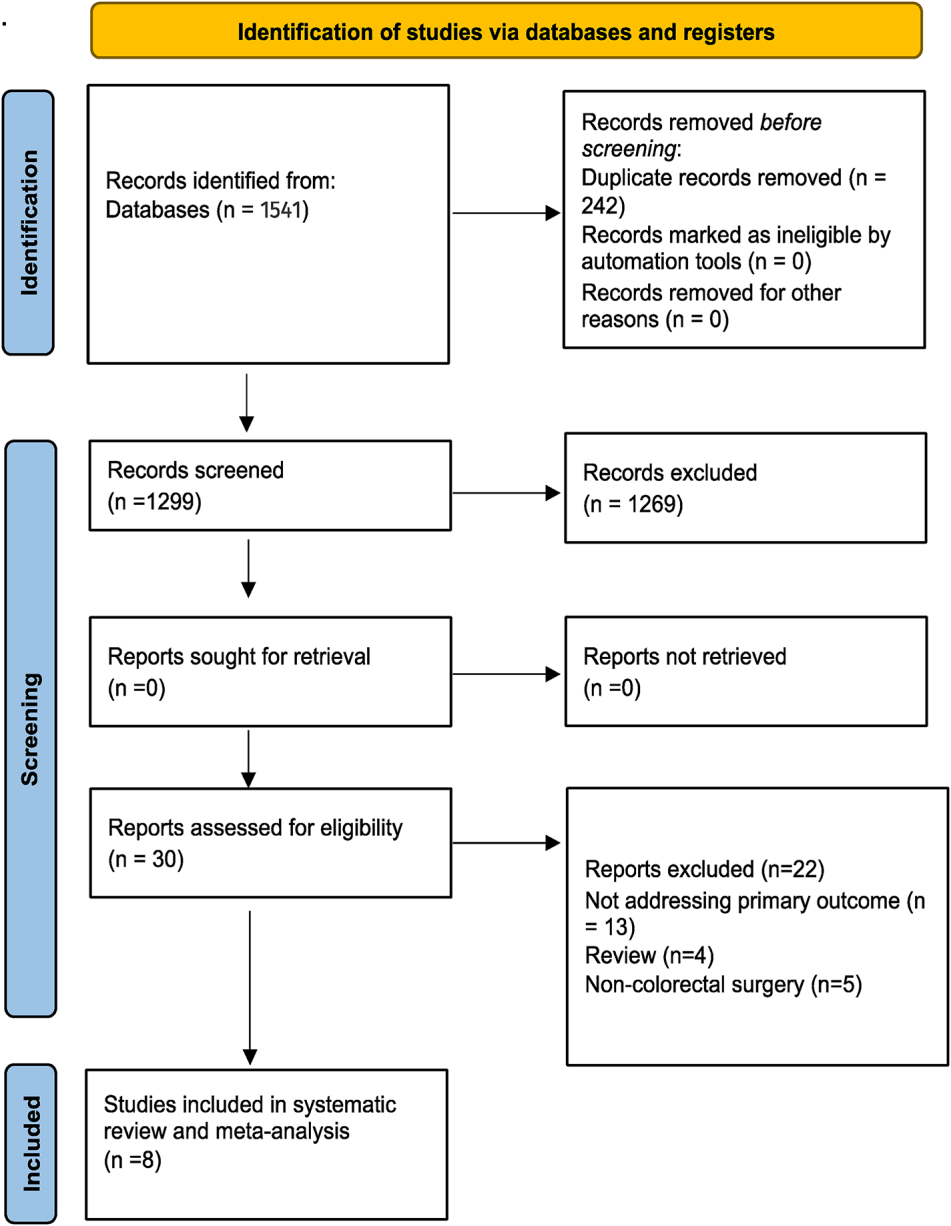
Preferred reporting items for systematic reviews and meta-analyses (PRISMA) flow chart (9).

**Figure 2:**
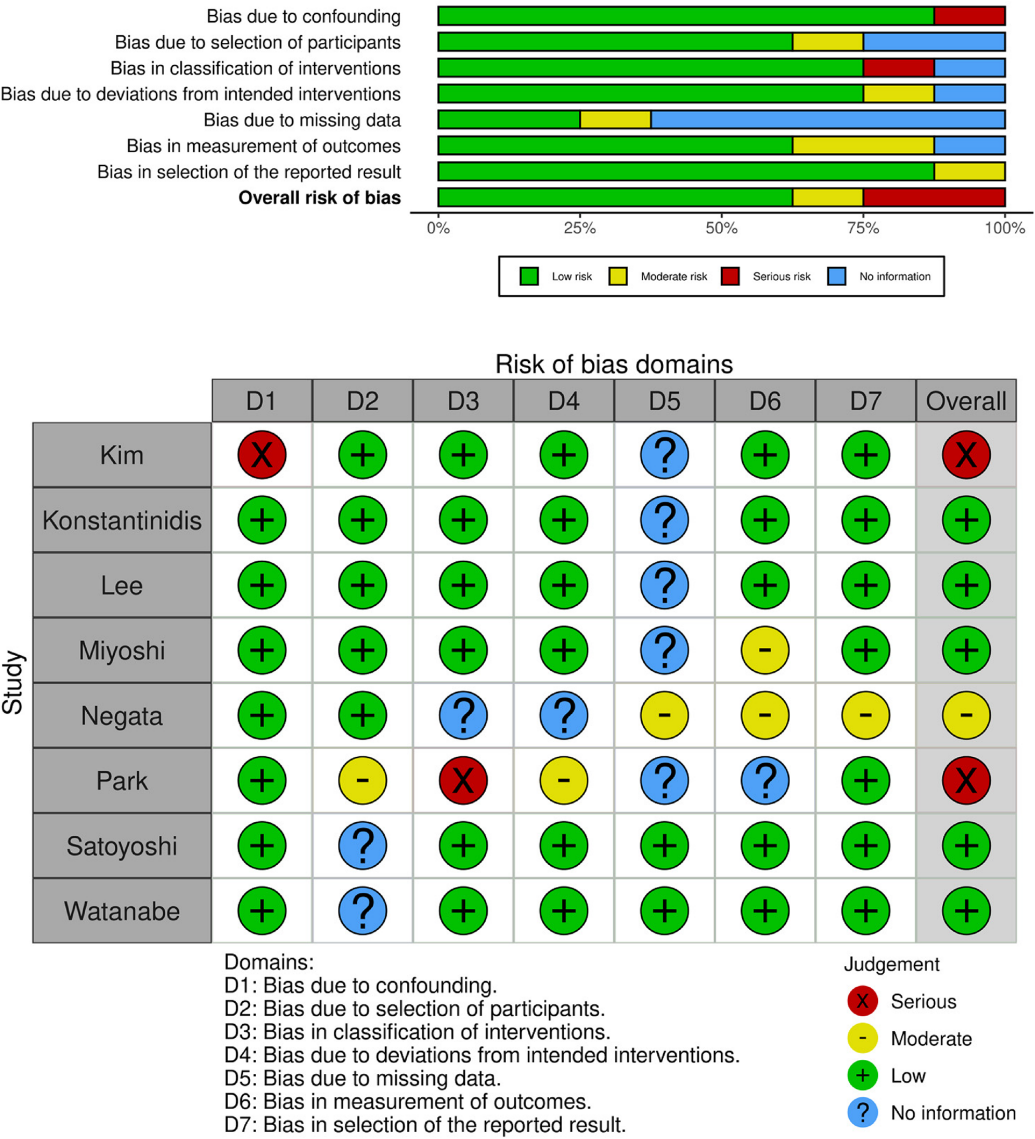
ROBINS-I tool for bias assessment of the included studies.

A database search identified 1,541 articles. After duplicates were removed, 1,299 articles remained. An analysis of titles and abstracts yielded 300 relevant articles for the full text review. Publications that met final inclusion criteria included 8 articles published between 2008 and 2022 (11–18). A total of 1,061 patients were included, of whom 696 were preoperatively tattooed with ICG. Figure 1 shows the study selection using the PRISMA flowchart. Four of the included studies were undertaken in Japan, three in South Korea, and one in Greece (Table 1). Overall, two studies were considered high risk of bias, one was moderate, whereas five were low risk, according to ROBINS-1 tool (Figure 2) (10). All of the studies were single center. These included two retrospective cohort studies, four prospective case-series studies, and two retrospective case-series. From the available reported data, a total of 201 patients (190 markings with ICG) underwent resection of the right colon (including transverse colon), 353 patients (294 markings with ICG) had an operation of the left colon, and 275 patients (257 markings with ICG) had a rectal tumor. Laparoscopic surgery was the most often used approach in a total of 937 patients, whereas an open operation was used in 75 patients and a robotic approach in 10 (Miyoshi et al. used both laparoscopic and open methods without the clarifying number of patients in each).

### Technique of preoperative ICG injection

Indocyanine green (25 mg) was diluted with sterile water or normal saline and the final solution ranged from 2.5–12.5 mg/ml. Injection dosage ranged from 0.1–1.5 ml of solution at either 1 site distal to the tumor, 2 sites 180° apart, 3 sites 120° apart (circumferentially) or at all 4 quadrants. The saline test injection method was used in four studies prior to injecting ICG into the submucosal layer of the colon. Using either a 23-gauge or a 26-gauge injection needle, 0.2–2 ml of saline was injected into 1 or 2 sites of the submucosal layer to form proper submucosal elevation. Using a second syringe and needle, ICG solution was injected. After injecting ICG into elevated submucosal layers, both Sang Lee et al. and Miyoshi et al. replaced the syringe with the first one and injected 1–2 ml normal saline, respectively, to flush out the remaining ICG in the needle. Two studies did not use the saline test injection method and injected ICG directly into submucosal layer. Watanabe et al. as well as Nagata et al. did not specify whether they employed this technique (Table 2).

### Marking-Detection rates

Patients were scheduled for preoperative colonoscopy and tumor marking on different days prior to surgery. In most of the included studies, the highest detection rates were achieved when the tattoo was performed within one week of surgery. More specifically, Satoyoshi et al. successfully detected all 141 (100%) tumors that were marked ≤6 days before surgery. Watanabe et al. presented similar results (75/76 tumors detected, 98.7%) in markings ≤7 days. Similarly, Miyoshi et al. described a 100% tattoo detection rate (28/28) within 8 days performed. When the preoperative colonoscopy was performed up to 3 days prior to the surgery, Kim et al. detected 85/90 (94.4%) of the tumor markings and Nagata et al. successfully found all 24 (100%) tumors intraoperatively. Sang Lee et al. reported 170/170 (100%) positive tumor detections after tattoo marking within 2 days, but only 2/5 (40%) after 2 days. Finally, when patients underwent colonoscopy one day prior to the operation, Park et al., Nagata et al. and Konstantinidis et al. detected all marked tumors [114/114 (100%), 24/24 (100%) and 10/10 (100%), respectively].

When the preoperative tattoo was performed more than one week prior to the operation, less tumors were detected. Satoyoshi et al. reported a 60% (6/10) successful rate in markings performed 7–9 days prior to the resection and 0% (0/4) when performed >10 days prior. Similarly, Watanabe et al. did not successfully detect any of the marked tumors in 4 patients (0/4, 0%) between 8 and 17 days prior to surgery. Finally, Miyoshi et al. observed positive staining in only 2/10 (20%) patients who underwent tumor marking ≥9 days prior to their operation day (Table 3). In total, when the marking was placed within one week, the visualization rate was 97% (650/668) whereas when it was longer than one week, the detection rate was 14% (8/5).

**Table 3:** Outcomes and conclusions.

Studies	Detection-rates	Complications	Conclusions
Satoyoshi et al.	a. ≤6 d: 141/141–100% b. 7–9 d: 6/10–60% c. ≥10 d: 0/4–0%	Some cases of spillage into serosa that obscured separation boundary in NIR view without disturbing view in white-light	ICG marking should be performed within at least 6 days prior of laparoscopic colorectal resections
Kim et al.	≤3 d: 85/90–94.4%	One patient with mild abdominal pain	Direct injection method with ICG without N/S elevation (saline test injection) can be used as alternative tattooing method for colorectal tumors when performed within 3 days prior of surgery
Sang Lee et al.	a. ≤2 d: 170/179–95% b. >2 d: 2/5–40%	None	Positive staining of water-soluble ICG tended to be weaker and fainter over time, finally dissipating without forming foreign material. These results support the proposition that ICG can be a safe option for endoscopic marking.
Watanabe et al.	a. ≤7 d: 75/76–98.7% b. 8–17 d: 0/4–0%	None	Using ICG with PINPOINT system for identifying colonic tumor sites was feasible without adverse effects during laparoscopic colorectal surgery
Jae Park et al.	≤1 d: 114/114–100%	One patient had mucosal edema One patient had intrabdominal leakage of ICG without inflammation	Tattooing has a significant effect of reducing amount of blood loss by preventing resection of unnecessary parts Tattooing was associated with shorter post-operative bowel recovery
Nagata et al.	≤3 d: 24/24–100%	None	Fluorescence imaging offers high sensitivity in determination of tumor location even if black or green staining cannot be seen in white light Double injection technique (2 sites of injection) instead of 4 quadrant technique is preferable due to reduced risk of spillage and leakage
Miyoshi et al.	a. ≤8: 29/29–100% b. >8: 2/10–20%	One patient had peritoneal ICG spillage	ICG as endoscopic marker can be reliably identified up to 8 days prior of colorectal resections
Konstantinidis et al.	≤1 d: 10/10–100%	None	Visualization of preoperatively marked tumors with ICG offers a great choice that could potentially be considered by surgeons before colorectal procedures. The intraoperative view of previously marked tissues under near-infrared light can be very clear with bright intensity. Separation of marked and unmarked tissues can be easily evaluated during laparoscopy and guide the surgeon throughout the operation.

ICG, Indocyanine green; N/S, Normal saline; NIR, Near Infra-Red; d, days.

### Complications

There were no severe complications related to tattooing in any of the included studies. Satoyoshi et al. described some cases (without defining the number) of spillage into serosa that obscured the separation boundary in a NIR view without disturbing the view in white-light. Kim et al. reported one patient with mild abdominal pain upon tattooing, but without any further preoperative findings and symptoms. Both Park et al. and Miyoshi et al. documented one patient each with peritoneal ICG spillage, but without inflammation. In addition, Park et al. observed one patient with mucosal edema (Table 3).

## Discussion

During minimally invasive surgery, identification of neoplasms has posed challenges due to the absence of tactile sensation. Consequently, surgeons must rely on visual assessment along with preoperative imaging to guide them in achieving a precise surgical resection. In complex cases, preoperative localization and marking of tumors by endoscopists hold vital importance as they provide surgeons with valuable anatomical guidance. Although extremely rare, positive longitudinal resection margins in colorectal surgery in cases where localization of the tumor cannot be verified intraoperatively, have negative implications in oncologic outcomes. The practice of endoscopic tattooing for detecting colorectal lesions has been documented as early as 1975 (19). Since then, it has been upgraded in many aspects and is one of the most popular approaches due to its accurate lesion localization and small tumor detection. Several substances have been used as markers including, methylene blue, indigo carmine, toluidine blue, and isosulfan blue, but only India ink and ICG were reportedly visible up to 48 h after injection (13, 20–22).

Lee et al. compared the duration of marking between ICG and India ink in a randomized animal study and concluded that India ink had better durability (23). However, India ink is a combination of several substances capable of inducing inflammatory reactions such as focal peritonitis, inflammatory pseudotumors, abscesses, and adhesions and for that reason, it is used under an autoclave and after dilution (15, 22). Depending on the technique, numerous complications have been reported using India ink (24–29). For instance, due to its permanent marking method, in the case of leakage, it interferes with the surgical view and detection of anatomical structures.

Conversely, ICG is transient rather than indelible (12) and has been safely used for more than 50 years (30–32). In 1993, Hammond et al. were the first to describe the use of ICG as a colorectal tumor marker instead of India ink (21). Since then, various techniques have been reported regarding preoperative colorectal tumor marking with ICG.

This systematic review included eight studies published between 2008 and 2022 that included a total of 1,061 patients. Included studies were both prospective and retrospective ones, providing a comprehensive evaluation of the utility of preoperative ICG tumor marking in colorectal surgery.

The utilization of ICG as a preoperative tumor marking agent in colorectal surgery offers numerous advantages in terms of enhanced visualization, precise tumor localization, and improved surgical outcomes.

Regarding the technique of preoperative ICG injection, the studies exhibited some variations in the dosage and administration method. The ICG solution, typically prepared by diluting 25 mg of ICG with sterile water or normal saline, ranged from 2.5 to 12.5 mg/ml. The injection dosage varied from 0.1 to 1.5 ml, depending on the number of sites and quadrants chosen for injection. Some studies employed the saline test injection method, which involved injecting saline into the submucosal layer before administering ICG. This technique aimed to achieve proper submucosal elevation for effective ICG delivery. However, not all studies clarified whether they utilized this method. The heterogeneity in injection techniques emphasizes the need for standardization and further investigation into the optimal dosage and injection methods.

The marking-detection rates demonstrated the effectiveness of preoperative ICG tumor marking in colorectal surgery. In general, the highest detection rates were achieved when the tattooing was performed within one week of surgery. Studies have consistently reported high detection rates, ranging from 94.4% to 100%, for tumor markings conducted within one week before the operation. However, when the preoperative colonoscopy and tumor marking were performed more than one week prior to surgery, the detection rates tended to decrease. The same findings were reported in 2009 by Watanabe et al., in 8 patients who underwent preoperative ICG tumor injection (33). Ahn et al. performed a prospective study that included 192 patients who underwent endoscopic submucosal ICG tattooing near the lesion 12–18 h before laparoscopic surgery for colorectal cancer. The aim of the study was to establish the optimal protocol for preoperative endoscopic submucosal ICG injection to perform fluorescence lymph node mapping, along with undisturbed fluorescent tumor localization and ICG angiography. The amounts of injected ICG were divided into 5 categories: >12, 1–12, 1–0.5, 0.5–0.3, and <0.3 mg. The authors concluded that the highest success rate was the dose of 0.5–1 mg (34). It is important to note that the optimal timing for tumor marking with ICG remains to be definitively determined. Further research is warranted to ascertain the ideal timing between marking and surgery to maximize detection rates and minimize the risk of false negatives.

Reported complications associated with preoperative ICG tumor marking were minimal. The reviewed studies reported no severe complications related to tattooing with ICG. Some cases of spillage into the serosa were observed, which occasionally obscured the separation boundary in the Near-Infrared Light (NIR) view but did not affect the view in white light. Additionally, mild abdominal pain and mucosal edema were reported in isolated cases, but these complications did not have significant clinical implications.

However, we should outline that body mass index (BMI) was not reported in the included studies, therefore any correlation between ICG-marked tumor identification and BMI cannot be made. We believe that BMI and intraperitoneal fat play important roles and can alter the efficacy of ICG. Moreover, another critical note is that tumor stage and detection rate were not correlated in any of the included studies.

Several other techniques have been employed for the detection of colorectal tumors, including computed tomography (CT) scans, CT colonography, preoperative barium enema, proctoscopy with stitch, as well as colonoscopy with metallic clipping (35–41). However, barium enemas have proven ineffective in visualizing small tumors (38, 39). The use of metallic clips for tumor localization presents challenges, as clips can be difficult to visualize and may migrate (35–37). Narihiro et al. compared technologies and reported on the safety and efficacy of NIR fluorescent clips, achieving a detection rate of 94.1% without encountering adverse effects associated with clip marking (40). Intraoperative colonoscopy can serve to identify gastrointestinal lesions, but it prolongs the overall operation time and can lead to intestinal distention, potentially limiting the surgical field (12, 35). An alternative approach utilizing autologous blood from the patient instead of a dye has also been described (41–43). However, additional blood collection is required and an accurate location may be difficult to find due to bleeding during surgery. Kim et al. employed 6–12 ml of autologous blood for endoscopic tattooing and reported a visualization rate of 92.2%, with three patients (5.9%) experiencing endoscopic adverse effects associated with the technique (41).

Our study has several limitations. Missing data may have led to an incomplete understanding of the subset(s) of patients may benefit from endoscopic tattooing. Moreover, the majority of the included studies did not report control groups of other tattooing agents or non-tattooed patients, which could have led to a more robust understanding of the value of this technique as well as its complications. Furthermore, the preoperative marking day varied between the studies and the exact day of injection was not clearly reported in every study, which could have led to more precise conclusions. Future large prospective cohorts or randomized studies are needed to better establish definitive conclusions regarding the effectiveness of ICG marking on tumor detection.

## Conclusion

The available literature on preoperative tumor marking with ICG in colorectal surgery demonstrates its potential as a valuable tool for enhancing surgical precision and improving outcomes. The reviewed studies consistently highlight the benefits of enhanced visualization, precise tumor localization, and increased detection rates. ICG fluorescence marking with NIR light is a reliable method for tumor site marking if ICG is injected into the submucosal layer around the tumor within one week before laparoscopic or robotic colorectal surgery. Further research is also needed to investigate its cost-effectiveness compared to traditional permanent dyes as well as potential combination strategies with other imaging modalities. Despite the limitations and challenges associated with ICG, its utilization in preoperative tumor marking in colorectal surgery shows great promise and warrants continued investigation to refine its implementation and maximize its benefits for patients.
