# Supplementary material for: Preoperative tumor marking with indocyanine green (ICG) prior to minimally invasive colorectal cancer: a systematic review of current literature
Source: Front Surg. 2023 Aug 11;10:1258343. doi: 10.3389/fsurg.2023.1258343 (PMC10453801; doi:10.3389/fsurg.2023.1258343)
Supplement: Supplementary file 1 [file Table1.docx]

Supplementary Material

**Preoperative Tumor Marking with Indocyanine Green (ICG) prior to Minimally Invasive Colorectal Cancer: a Systematic Review of current literature**

**Michael K. Konstantinidis^1,2*^, Argyrios Ioannidis^1^, Pantelis Vasiliou^2^, Nikolaos Arkadopoulos^2^, Ioannis S. Papanikolaou^3^ , Konstantinos Stavridis^4^, Dimitrios Karagiannis^5^ , Manish Chand^6^ , Steven D. Wexner^7^ and Konstantinos Konstantinidis^1^**

^1^Department of General, Laparoscopic, Oncologic and Robotic Surgery, Athens Medical Center, Athens, Greece

^2^Fourth Department of Surgery, Attikon University Hospital, National and Kapodistrian University of Athens School of Medicine, Athens, Greece

^3^Hepatogastroenterology Unit, Second Department of Internal Medicine – Propaedeutic, Medical School, National and Kapodistrian University of Athens, Attikon University General Hospital, Athens, Greece

^4^2^nd^ Department of Obstetrics and Gynaecology, Aretaieion Hospital, University of Athens, Athens, Greece.

^5^Department of Gastroenterology and Hepatology, Athens Medical Center, Athens, Greece

^6^UCL Division of Surgery and Interventional Sciences, WEISS Centre, University College London, London, United Kingdom,

^7^Department of Colorectal Surgery, Ellen Leifer Shulman and Steven Shulman Digestive Disease Center, Cleveland Clinic Florida, Weston, FL 33331, USA.

*** Correspondence:**

Michael K. Konstantinidis

mikekonstantinidis@gmail.com

Keywords: Fluorescence imaging · Indocyanine green · Colorectal surgery · Colorectal cancer · Colorectal tumor · Preoperative tumor marking · Preoperative tattoo

**Table 1: Characteristics of included studies**

| Study | Country, Year | Study Type | Number of Patients (ICG use) | Operation Type (patients) | Primary Endpoint | ROBINS-I |
| --- | --- | --- | --- | --- | --- | --- |
| Satoyoshi et al | Japan, 2020 | Prospective case-series | 165 (165) | Laparoscopic Colorectal Resections (165) | Intraoperative detection rate of ICG marking |  |
| Kim et al | South Korea, 2020 | Retrospective cohort | 227 (90) | Laparoscopic Colorectal Resections (152)  Open (75) | Compare efficacy and safety between direct injection method with ICG and saline injection method with India Ink |  |
| Sang Lee et al | South Korea, 2018 | Retrospective case-series | 174 (174)  tattoo sites:184 | Laparoscopic Colorectal Resections (174) | 1; Usefulness of preoperative colonoscopic ICG tattoo with N/S elevation  2: Visualization rates in different sites of colon that are marked within 2 days prior of surgery |  |
| Watanabe et al | Japan, 2017 | Prospective case-series | 80 (80) | Laparoscopic Colorectal Resections (80) | Visibility of PINPOINT system for intraoperative identification of marked tumors with ICG |  |
| Jae Park et al | South Korea, 2018 | Retrospective cohort | 342 (114) | Laparoscopic Colorectal Resections (342) | 1; Usefulness of preoperative colonoscopic ICG tattoo in general prior of colorectal resections  2: Usefulness of preoperative colonoscopic ICG tattoo in groups classified according to stage and type of surgery |  |
| Nagata et al | Japan, 2016 | Prospective case-series | 24 (24) | Laparoscopic Colorectal Resections (24) | Feasibility and safety of imaging method using LED-activated ICG fluorescence for laparoscopic colorectal surgery |  |
| Miyoshi et al | Osaka, 2008 | Retrospective case-series | 39 (39) | Laparoscopic Colorectal Resections (N/A)  Open Colorectal Resections (N/A) | Timing of ICG tattoo prior of colorectal resections |  |
| Konstantinidis et al | Athens, 2022 | Prospective case-series | 10 (10) | Robotic Colorectal Resections (10) | Usefulness of preoperative colonoscopic ICG tattoo prior of robotic colorectal resections |  |

*ICG: Indocyanine green. N/S: Normal saline*

**Table 2: Procedure characteristics of studies**

| Study | ICG Solution (mg/ml) | N/S elevation | Injection Dosage (ml) | Injection Sites | Injection Time (days) | Groups of Patients | Tumor Location or Marking Sites |
| --- | --- | --- | --- | --- | --- | --- | --- |
| Satoyoshi et al | 5 | Yes (0,2ml-2 sites) | 0,1 | 2 | ≤6  7-9  ≥10 | Day of marking prior of operation: patients  a. ≤6: 141  b. 7-9: 10  c. ≥10: 4 | Right colon: 41  Left colon: 55  Rectum: 69 |
| Kim et al | N/A | No | 0,5 | 3 (circumferential) | ≤3 | Marked with ICG or India Ink: patients  a. India Ink: 79  b. ICG: 149 | Right colon: 53 (ICG:42)  Left colon: 127 (ICG: 68)  Rectum: 57 (ICG: 39) |
| Sang Lee et al | N/A | Yes (1ml-1 site) | 1-1,5 | 2, 180° apart or  3, 120° apart | 0  1  2  3-14 | Day of marking prior of operation: tattoo sites  a. ≤2: 179  b.>2: 5 | Ascending colon: 7  Hepatic flexure: 11  Transverse colon: 20  Splenic flexure: 5  Descending colon: 12  Sigmoid: 87  Rectosigmoid: 26  Rectum: 16 |
| Watanabe et al | 2,5 | N/A | 0,5 | N/A | 1  3  5  6  7  8-10 | Day of marking prior of operation: patients  a. ≤7: 76  b. 8-17: 4 | Right colon: 19  Left colon: 27  Rectum: 34 |
| Jae Park et al | 12,5 | Yes (1-2ml) | 0,5-1 | 4 | 1 | Marked with ICG or India Ink: patients  Yes: 114  No: 228 | Right colon: 25  Left colon: 10  Rectum: 53 |
| Nagata et al | 2,5 | N/A | 0,5 | 1 site with ICG  1 site with India Ink | ≤3 | Single group of patients with simultaneous tattoo with ICG and India Ink | Ascending colon: 6  Transverse colon: 3  Descending colon: 3  Sigmoid: 4  Rectosigmoid: 5  Rectum: 3 |
| Miyoshi et al | 12,5 | Yes (2ml-1 site) | 1 ml | 2 | 1-73 (median:4) | Day of marking prior of operation: patients  a. ≤8: 29  b. >9: 10 | Cecum: 1  Ascending colon: 8  Transverse colon: 6  Descending colon: 2  Sigmoid: 15  Rectosigmoid: 5  Upper Rectum: 3  Lower Rectum: 1 |
| Konstantinidis et al | 2,5 | No | 0,1 | 2 | 1 | Single group of patients marked with ICG | Right colon: 1  Left colon: 6  Rectum: 3 |

*ICG: Indocyanine green. N/S: Normal saline*

**Table 3: Outcomes and Conclusions**

| **Studies** | Detection-Rates | Complications | Conclusions |
| --- | --- | --- | --- |
| Satoyoshi et al | a. ≤6d: 141/141-100%  b. 7-9d: 6/10-60%  c. ≥10d: 0/4-0% | Some cases of spillage into serosa that obscured separation boundary in NIR view without disturbing view in white-light | ICG marking should be performed within at least 6 days prior of laparoscopic colorectal resections |
| Kim et al | ≤3d: 85/90-94.4% | One patient with mild abdominal pain | Direct injection method with ICG without N/S elevation (saline test injection) can be used as alternative tattooing method for colorectal tumors when performed within 3 days prior of surgery |
| Sang Lee et al | a. ≤2d: 170/179-95%  b. >2d: 2/5-40% | None | Positive staining of water-soluble ICG tended to be weaker and fainter over time, finally dissipating without forming foreign material. These results support the proposition that ICG can be a safe option for endoscopic marking. |
| Watanabe et al | a. ≤7d: 75/76-98.7%  b. 8-17d: 0/4-0% | None | Using ICG with PINPOINT system for identifying colonic tumor sites was feasible without adverse effects during laparoscopic colorectal surgery |
| Jae Park et al | ≤1d: 114/114-100% | One patient had mucosal edema  One patient had intrabdominal leakage of ICG without inflammation | Tattooing has a significant effect of reducing amount of blood loss by preventing resection of unnecessary parts  Tattooing was associated with shorter post-operative bowel recovery |
| Nagata et al | ≤3d: 24/24-100% | None | Fluorescence imaging offers high sensitivity in determination of tumor location even if black or green staining cannot be seen in white light  Double injection technique (2 sites of injection) instead of 4 quadrant technique is preferable due to reduced risk of spillage and leakage |
| Miyoshi et al | a. ≤8: 29/29-100%  b. >8: 2/10-20% | One patient had peritoneal ICG spillage | ICG as endoscopic marker can be reliably identified up to 8 days prior of colorectal resections |
| Konstantinidis et al | ≤1d: 10/10-100% | None | Visualization of preoperatively marked tumors with ICG offers a great choice that could potentially be considered by surgeons before colorectal procedures. The intraoperative view of previously marked tissues under near-infrared light can be very clear with bright intensity. Separation of marked and unmarked tissues can be easily evaluated during laparoscopy and guide the surgeon throughout the operation. |

*ICG: Indocyanine green. N/S: Normal saline. NIR: Near Infra-Red d: days*

Figure 1. Preferred Reporting Items for Systematic Reviews and Meta-Analyses (PRISMA) flow chart [9]

**
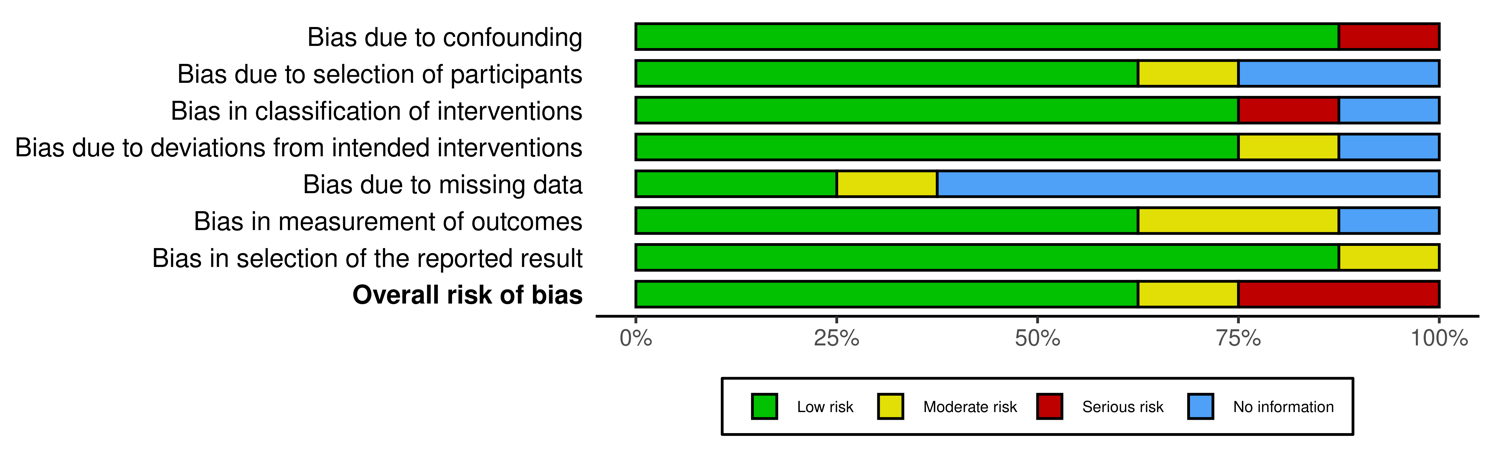
**


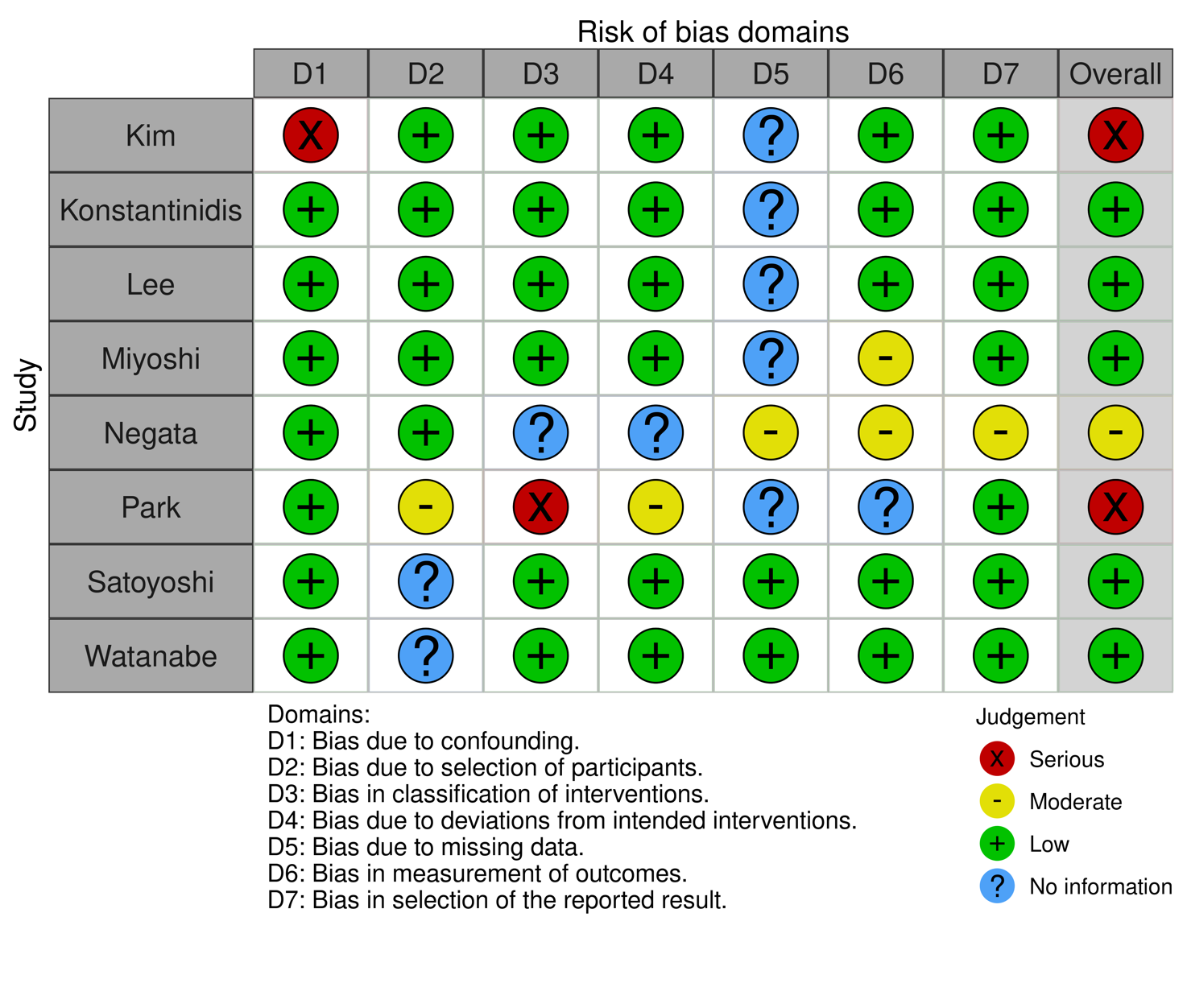


Figure 2. ROBINS-I tool for bias assessment of the included studies.
